# Seasonal and phenotypic effects on sperm quality in a taurine breed acclimated to the tropics versus a specialized commercial Zebu breed

**DOI:** 10.1007/s11250-026-05019-4

**Published:** 2026-04-17

**Authors:** Paula Lorena Grangeira Souto, Eleonora Araújo Barbosa, Raquel Soares Juliano, Luciana Keiko Hatamoto-Zervoudakis, Connie McManus, Ernandes Rodrigues de Alencar, Rodrigo Dorneles Tortorella, Alexandre Floriani Ramos

**Affiliations:** 1 Universidade de Brasília, Brasília, 70910-900 Brazil; 2 Embrapa Pantanal, Corumbá, 79320-900 Brazil; 3 Universidade Federal do Mato Grosso, Cuiabá, 78060-900 Brazil; 4 Universidade Estadual do Centro-Oeste, Guarapuava, 85015-430 Brazil; 5 Embrapa Recursos Genéticos e Biotecnologia, Parque Estação Biológica, Av. W5 Norte (Final), Brasília, 70770-917 Brazil

**Keywords:** Adaptation, Food security, Heat stress, Genetic resources, Livestock, Reproduction

## Abstract

Heat stress is a growing challenge for cattle production in tropical environments, particularly for *Bos taurus* breeds, which are generally less heat‑tolerant. Several locally adapted breeds have evolved morphological and physiological traits that combine characteristics of both *Bos taurus* and *Bos indicus* subspecies. We evaluated seasonal changes in semen quality and their association with environmental conditions and external body morphology in Pantaneiro (locally adapted *Bos taurus*) and Nelore (*Bos indicus*) bulls in the Brazilian Pantanal. Environmental conditions differed markedly between seasons, with the rainy season presenting a higher discomfort index. During the dry season, the index ranged from comfort to danger (45–83), whereas in the rainy season it ranged from alert to emergency (74–84). Fresh semen quality showed no major seasonal or breed differences, with motility averaging around 83% in both groups. In contrast, post-thaw sperm kinematic parameters, evaluated using the computer-assisted sperm analysis (CASA) system, showed clear breed differences. Pantaneiro bulls had higher motile sperm (43% vs. 31% in the dry season; 45% vs. 34% in the rainy season; *P* = 0.04), progressive sperm (26% vs. 17% in the dry season; 23% vs. 16% in the rainy season; *P* = 0.02), and the proportion of spermatozoa classified as rapid by CASA (32% vs. 20% in the dry season; 30% vs. 21% in the rainy season; *P =* 0.01) compared to Nelore bulls. Average path velocity (VAP) and straight-line velocity (VSL) were also higher in Pantaneiro bulls, particularly during the dry season. The viability of post-thaw sperm membranes and chromatin integrity followed the same pattern: the proportion of cells with intact acrosome, intact plasma membrane and high mitochondrial membrane potential was higher in Pantaneiro (41% in the dry season, 52% in the rainy season) than in Nelore bulls (34% in both seasons). External morphological traits, such as body size, skinfold thickness, hair length and skin color, were negatively correlated with spermatic kinetics and membrane integrity (*r* ≤ − 0.30). Overall, Pantaneiro bulls outperformed Nelore bulls under the same conditions. Both breeds maintained acceptable semen quality under heat stress, but Pantaneiro bulls showed more stable and superior post-thaw performance. These results reinforce the conservation value of this genetic resource. As a *Bos taurus* well adapted to tropical environments, it is an important resource for breeding programs targeting resilience and reproductive efficiency.

## Introduction

According to FAO (2022a), 17% of local breeds of livestock are at risk of extinction and cattle are the species with the highest number of breeds classified as extinct. Furthermore, 58% of breeds are classified as having unknown risk due to the lack of recent registration or evidence of existence. Gene banks are strategic tools for strengthening in situ conservation and serve as a backup to secure biodiversity for food and agriculture and for genetic improvement programs (Mariante et al. 2009, 2011; FAO, 2022a,b). Besides, there is substantial variation in post-thawing semen viability linked to several phenomena such as genetics, health, semen processing, and other endogenous and exogenous factors (Dementieva et al. 2024; Umirbaeva et al. 2024). In tropical countries, such as Brazil, heat stress may alter sperm parameters, including sperm abnormalities, membrane integrity, sperm kinematics, and DNA denaturation, which are detectable only about two weeks after stress. This is an important concern given the prevalence of extensive livestock production systems. Therefore, bulls’ capacity to maintain homeostasis plays a key role in preserving semen quality (Freitas et al. 2020; Llamas-Luceño et al. 2020).

Routine evaluation of semen quality in andrological practice is based on a combination of complementary parameters that reflect sperm functionality and fertilizing potential (Amann and Waberski 2014; Mortimer et al., 2015). Sperm kinematic parameters obtained by computer-assisted sperm analysis (CASA), such as velocity and motility patterns, are widely used as indicators of sperm vigor and transport capacity within the female reproductive tract (Mortimer et al., 2015). In addition, assays of plasma membrane integrity provide information on cell viability and the ability to maintain homeostasis, whereas chromatin integrity assessments reflect DNA stability and are directly associated with embryo development and fertility outcomes (Aitken and De Iuliis 2010; Evenson 2016). In the context of artificial insemination and germplasm conservation, particularly under tropical conditions, post-thaw semen evaluation is essential, as cryopreservation may exacerbate sublethal damage caused by heat stress, compromising sperm function even when conventional parameters appear normal (Watson 2000).

The Pantaneiro is a *Bos taurus* breed that evolved for at least three centuries in the Brazilian Pantanal and has acquired adaptive traits to withstand the harsh environmental conditions of this region, which experiences an annual cycle of floods (Juliano et al. 2022). Due to its ability to maintain homeostasis and adapt to the Pantanal’s climatic conditions, the breed produces milk with high fat content (Oliveira-Brochado et al. 2018) and tender meat (Barbosa et al. 2023). The breed played an important role in the economy of the Pantanal region until the early 20th century. More recently, Pantaneiro cattle are at risk of extinction due to its gradual replacement by specialized exotic breeds such as Nelore (Egito et al. 2002).

Despite the conservation relevance of locally adapted breeds, data on how seasonal climatic variation affects semen quality, particularly in comparison with commercial breeds, remain scarce. Thus, this study aimed to analyze the effect of seasons on fresh and post-thawing semen quality of locally adapted Pantaneiro (*Bos taurus*) bulls compared to specialized Nelore (*Bos indicus*) bulls in Brazilian Pantanal, considering the influence of phenotype of the animals. Based on previous evidence of better thermotolerance in locally adapted breeds, we hypothesized that seasonal changes in climate would affect semen quality in both breeds, but Pantaneiro bulls would show comparatively better performance due to their adaptation to tropical environments.

## Materials and methods

### Animals and experimental design

This study was carried out at Nhumirim Experimental Farm, owned by Embrapa Pantanal, an ecoregional research unit of the Brazilian Agricultural Research Corporation (EMBRAPA), located in Southern Pantanal, Nhecolândia sub-region, Corumbá, Mato Grosso do Sul, Brazil (18° 59’ 19.9” S, 56° 37’ 21.7” W). The predominant climate is the tropical continental, characterized by two well-defined seasons: a rainy summer and a dry winter. The farm has 4,390.6 ha and represents 19.48% of the total Pantanal area.

Twelve healthy adult bulls were included in the study: six Nelore (5.1 ± 1.5 years) and six Pantaneiro (4.8 ± 0.8 years). All animals fell within the expected adult age range for the species. The bulls were considered reproductively sound based on a breeding soundness evaluation and had been separated from the cow herd for at least 15 days. Pasture consisted of *Brachiaria decumbens*, *B. humidicola*, and native grass of Capim-carona (*Elyonurus muticus*), Capim-de-capivara (*Hymenachne amplexicaulis*), and Grama-do-carandazal (*Panicum laxum*), with *ad libitum* water and mineral supplementation.

### Climatic data

Air temperature (AT, °C) and relative humidity (RH, %) data were obtained daily starting at 35 days before the first semen collection by an automated meteorological station located on the farm. Mean radiant temperature (MRT, °C) was measured three times a day using a portable black globe thermometer (Reis et al. 2021). The Temperature-Humidity Index (THI) was calculated according to Thom’s formula (Thom 1959), as follows:

$$\:\mathrm{T}\mathrm{H}\mathrm{I}\:=\:(0.8\:\times\:\:\mathrm{A}\mathrm{T})\:+\:\left[\right(\mathrm{R}\mathrm{H}\:/\:100)\:\times\:\:(\mathrm{A}\mathrm{T}\hspace{0.17em}-\hspace{0.17em}14.4\left)\right]\hspace{0.17em}+\hspace{0.17em}46.4$$

where AT is air temperature (°C) and RH is relative humidity (%). THI values were classified into four zones as “Comfort zone” (61–74), “Alert zone” (75–78), “Danger zone” (79–83), and “Emergency zone” (≥ 84) (Thom 1959; Hahn 1985; Mader et al. 2006).

### Morphometric evaluation

Measurements of body weight, body condition score (BCS), withers height, croup height, body length, chest circumference, chest depth, shin perimeter, scrotal circumference and testicular biometrics (testicular thickness, length and width) were obtained in both seasons. The testicular volume (TV) was calculated using the following formula:

$$TV{\mkern 1mu} = {\mkern 1mu} 2 \times \left[ {{{\left( {TW/2} \right)}^2} \times 3.14 \times \left( {TL} \right)} \right]$$

where TV is the testicular volume (cm³), TW is the testicular width (cm) and TL is the testicular length (cm) (CBRA 2013).

The thickness of the skin fold of the testicle was measured with a caliper on the lateral surface of the testicle. Hair coat density and hair coat length were obtained following Udo (1978) and Silva (2000). A hair sample was collected from the same area of the lateral thoracic region using adapted pliers designed to extract a standardized 1 cm² patch. All hairs removed within this defined area were placed on grid paper, counted manually and measured individually to determine hair density (hairs/cm²) and mean hair length. The percentage of epidermal pigmentation was assessed visually using a standardized printed scale for cattle ranging from 0 to 100, based on the intensity of black to red pigmentation (Silva 2000).

The color of the hair coat was measured in triplicate using the CIELAB system method (ColorQuest XE Spectrophotometer, HunterLab, Reston, USA). This system determines three colorimetric coordinates, L*, a*, and b*, which can assume positive or negative values. The L* coordinates represent the lightness (near to 100) and darkness (near to 0); Positive a* values indicate red tones, whereas negative values indicate green tones. Positive b* values indicate yellow tones, whereas negative values indicate blue tones. The hue angle (H*) was calculated using the arctan2 function applied to the a* and b* components, following the formula:

$${\rm{H*}} = {\rm{MOD}}\left({{\rm{arctan}}2\left({{\rm{b*}},{\rm{a*}}} \right),{\rm{}}360{\rm{}}} \right)$$

These variables follow the standard notation of the CIELAB color system, where the asterisk (*) is part of the coordinate name and does not denote statistical significance. The resulting values were adjusted to a continuous 0° to 360° scale (Weatherall and Coombs 1992).

### Semen collection

Semen was collected by electroejaculation (Duboi^®^ electroejaculator, Campo Grande, MS, Brazil) on three different days per season, with a three-day interval between collections, following Menegassi et al. (2015). Samples were kept at 37 °C until further analysis. Twelve bulls (six Pantaneiro and six Nelore) were evaluated; each bull provided three ejaculates per season (one ejaculate per collection day), yielding 18 ejaculates per breed per season. Ejaculate volume, color, and appearance were evaluated during the initial andrological examination to confirm reproductive soundness before the experiment began, and only bulls with normal baseline ejaculate traits were included. The individual bull was defined as the experimental unit.

### Laboratory analyses

After semen collections, fresh semen was conventionally evaluated by a single evaluator for sperm motility (%), vigor (0–5) and sperm concentration using a Neubauer chamber (x 10^9^ sptz/mL) (Teixeira et al. 2011; Souto et al. 2017). Sperm morphology was evaluated by phase contrast light microscopy. Briefly, 60 µL of semen were fixed in 1 mL of buffered formal saline, and 200 cells were examined and classified as normal, major defects or minor defects according to standard criteria (CBRA 2013; Moreira et al. 2016).

For semen freezing, the ejaculates were diluted to a concentration of 40 × 10^6^ sperm per 0.5 mL straw (French straws, Minitube, Tiefenbach, Germany) in a Tris egg yolk-based extender (Triladyl^®^, Minitube, Tiefenbach, Germany) supplemented with 20% (v/v) fresh egg yolk). The extended semen was packaged and frozen using a programmable freezing machine (TK-3000^®^, TK Freezing technology Ltda, Uberaba, Brazil). The cooling curve started at − 0.5 °C/min, 45 min, followed by an equilibrium phase at 5 °C, 4 h, ending at a freezing curve of − 20 °C/min until reaching − 120 °C when the straws were immersed in liquid nitrogen and stored in a cryogenic device.

Three straws per batch were thawed at 37 °C for 30s in a water bath and analyzed for sperm abnormalities, membrane integrity, sperm kinematics, and DNA denaturation, then Computer-assisted semen analysis (CASA) was used for kinematic analysis. The CASA (Sperm analysis System, IVOS-Ultimate 12’s, Hamilton Thorne Biosciences, Beverly, MA, USA) was used to evaluate post-thaw sperm kinematics. The CASA setup was adjusted for bovine sperm analysis, as follows: number of frames required: 30; frame rate: 60 Hz; minimum contrast: 60 pixels; minimum cell size: 5 pixels; straightness (STR) cut-off: 70%; VAP cut-off: 30 μm/s; VSL cut-off: 20 μm/s; magnification: 1.95×. A drop of 10 µL of thawed semen was placed in a pre-warmed (37 °C) glass slide (Makler^®^ chamber, Sefi Medical Instruments, Haifa, Israel, 10 μm) and loaded into the equipment. Five fields were randomly selected for examination of the motile and progressive cells, VAP, VSL, curvilinear velocity (VCL), amplitude of lateral head displacement (ALH), beat cross frequency (BCF), STR, linearity (LIN), and slow, rapid and static cells.

The staining protocols followed Klinc and Rath (2007), Harrison and Vickers (1990), Celeghini et al. (2010), and Unanian (2000), with minor adaptations. Two hundred spermatozoa were evaluated under a fluorescence microscope using a green filter at 1000× magnification with an oil-immersion objective.

Acrosomal integrity was evaluated using fluorescein isothiocyanate-conjugated peanut agglutinin - FITC-PNA (Sigma-Aldrich^®^, L7381) and propidium iodide – PI (Molecular Probes^®^, P1304) as described before (Souto et al. 2022). Briefly, for the FITC-PNA/PI assay, FITC-PNA was used at 1 mg/mL and PI at 0.5 mg/mL. Ten microliters of semen were incubated with 40 µL of the working solution for 10 min at room temperature in the dark. Cells with intact plasma membranes were PI-negative, and cells with damaged plasma membranes were PI-positive. Reacted acrosomes (FITC-PNA positive) exhibited a green fluorescence, whereas intact acrosomes (FITC-PNA negative) were nonfluorescent.

Plasma membrane status was assessed using 6-Carboxyfluorescein diacetate - CFDA (Sigma-Aldrich^®^, C-5041) with PI according to the method described by Harrison and Vickers (1990). Intact cells stained green, whereas damaged cells stained red.

Simultaneous assessment of sperm membranes was performed using a combination of FITC-PNA, PI, and JC-1 (5,5′6,6′ -Tetrachloro-1,1′ 3,3′ -tetraethyl-imidacarbocyanine iodide, Molecular Probes^®^, T-3168) followed the method described before (Souto et al. 2022). Briefly, the triple staining (FITC-PNA, PI, JC-1) used 1 µL FITC-PNA, 2 µL PI, and 5 µL JC-1 (153 µM stock) added to 40 µL of semen diluted in TALP and incubated for 8 min at 37 °C. Sperm were classified into eight categories according to fluorescence patterns, as shown in Table 1.

**Table 1:** Sperm cell categories according to fluorescence emitted in the triple-staining protocol

Category	Description	FITC-PNA^a^	PI^b^	JC-1^c^
JC-1a	Intact acrosome, intact plasma membrane and high mitochondrial membrane potential	–	–	orange
JC-1b	Reacted acrosome, damaged plasma membrane and low mitochondrial membrane potential	green	red	green
JC-1c	Intact acrosome, damaged plasma membrane and low mitochondrial membrane potential	–	red	green
JC-1d	Intact acrosome, intact plasma membrane and low mitochondrial membrane potential	–	–	green
JC-1e	Intact acrosome, damaged plasma membrane and high mitochondrial membrane potential	–	red	orange
JC-1f	Reacted acrosome, damaged plasma membrane and high mitochondrial membrane potential	green	red	orange
JC-1 g	Reacted acrosome, intact plasma membrane and low mitochondrial membrane potential	green	–	green
JC-1 h	Reacted acrosome, intact plasma membrane and high mitochondrial membrane potential	green	–	orange

^a^FITC-PNA: acrosome membrane probe; ^b^PI: plasma membrane probe; ^c^JC-1: mitochondrial membrane potential probe

Chromatin integrity was assessed using the acridine orange (AO) assay, as described by Unanian (2000). Abnormal chromatin displayed an orange-red fluorescence (denatured, single-stranded DNA), whereas normal chromatin displayed a green fluorescence (native DNA).

### Statistical analysis

All analyses were conducted in SAS (Version 3.81, Enterprise Edition; SAS Institute Inc., Cary, NC, USA), which was also used to generate the figures. Significance threshold was set at *P* < 0.05. Data distribution was examined with the UNIVARIATE procedure. Normality of residuals was assessed using the Shapiro–Wilk test, supported by Kolmogorov–Smirnov, Cramer–von Mises and Anderson–Darling goodness-of-fit statistics. Homogeneity of variances was evaluated using Levene’s test.

Because several response variables did not fully meet normality and homoscedasticity assumptions and considering the repeated-measures structure of the data, generalized linear mixed models were fitted using the GLIMMIX procedure. Residual distributions and link functions were specified according to the scale and empirical distribution of each response variable. Continuous traits with approximately normal distribution were modeled assuming a Gaussian distribution with identity link. Proportional variables were analyzed using a binomial distribution with logit link. Continuous traits with strictly positive and markedly skewed distributions were modeled using a gamma distribution with log link, following commonly recommended specification for such outcomes in generalized linear mixed models.

The individual bull was defined as the experimental unit. Season, breed and their interaction were included as fixed effects, and bull was modeled as a random effect to account for repeated measurements and avoid pseudo-replication. Pairwise comparisons among least-square means were performed using LSMEANS statement with the PDIFF option and Tukey–Kramer adjustment to control the family-wise type I error rate. Results are presented as mean ± standard deviation (SD).

Spearman correlations were estimated with the CORR procedure to assess the strength and direction of associations among the variables. Interpretation focused on correlations of at least moderate magnitude (|r| ≥ 0.30), following Cohen’s effect-size criteria. Principal component analysis (PCA) was run separately for each season using the PRINCOMP procedure. The number of components retained was determined by selecting the smallest set of principal components that together accounted for at least 70% of the total variance.

## Results

### Environmental variables

Significant daily variations were observed for environmental variables in both seasons, but the ranges were larger in the dry season (Table 2). All variables changed significantly between periods of the day and seasons, except for MRT.

**Table 2:** Summary statistics (mean ± SD) for environmental variables in dry and rainy season at Nhumirim experimental farm

Parameters		Dry season		Rainy season	
		Morning	Afternoon	Morning	Afternoon
AT (°C) (*n* = 420)	Mean ± SD	17.3 ± 6.8^c^	28.0 ± 5.4^a^	25.4 ± 1.0^b^	28.9 ± 2.6^a^
	Min - Max	6.7–28.4	15.7–37.4	24.9–25.6	28.0–29.3
RH (%) (*n* = 420)	Mean ± SD	83.1 ± 10.6^b^	38.3 ± 7.3^d^	92.6 ± 4.5^a^	73.2 ± 10.8^c^
	Min - Max	63.0–96.0	26.0–70.0	91.9–94.6	70.8–78.3
THI (*n* = 420)	Mean ± SD	62.2 ± 11.2^c^	74.0 ± 6.7^b^	76.9 ± 1.3^ab^	80.1 ± 2.5^a^
	Min - Max	44.7–78.3	65.3–83.2	74.1–79.7	76.6–83.9
MRT (°C) (*n* = 105)	Mean ± SD	37.0 ± 7.7	37.7 ± 9.2	36.7 ± 8.9	40.2 ± 9.2
	Min - Max	21.3–49.2	21.3–50.8	25.8–49.5	29.1–54.7

AT: Air temperature; RH: relative humidity; THI: Temperature-humidity Index; MRT: Mean radiant temperature; SD: standard deviation. AT and RH were obtained hourly from the Nhumirim Farm weather station. Means within a row with different lowercase letters differ significantly at *P* < 0.05

The analysis of THI data revealed that during the dry season, the index varied from the “Comfort zone” to the “Alert zone” in the morning, reaching the “Danger zone” in the afternoon. During the rainy season, the index varied from “Alert zone” in the morning to “Danger zone” in the afternoon, reaching “Emergency zone” in the afternoon.

Even during the dry season, when pasture was less available, all bulls maintained an ideal body condition score (BCS), as assessed by visual inspection of the rump, ribs, chest, and tail insertion.

### Morphometric traits

Morphometric measurements and coat traits were influenced by breed and season (*P* < 0.05). The hair density of Nelore bulls varied significantly across seasons, whereas that of Pantaneiro bulls did not. Pantaneiro bulls were smaller (*P* < 0.05) than Nelore bulls and, as a result, had a larger surface area-to-volume ratio (*P* < 0.05) (Table 3). Scrotal skinfold thickness and body skinfold thickness were thinner in Pantaneiro bulls (*P* < 0.05). Looking at the hair coat and skin traits, Pantaneiro bulls were darker, and Nelore bulls had the most reflective hair coat. All bulls had shorter and slightly denser hair coats in the rainy season.

**Table 3:** Morphological traits (mean ± SD) of Pantaneiro and Nelore bulls in dry and rainy season

Parameters	Dry season		Rainy season		P-Breed	*P*-Season	*P*-Breed*Season
	Nelore (*n =* 6)	Pantaneiro (*n =* 6)	Nelore (*n =* 6)	Pantaneiro (*n =* 6)			
BW (kg)	728.7 ± 63.9	519.8 ± 61.4	704.3 ± 58.3	505.2 ± 63.4	<0.0001	0.4753	0.8578
BCS (1–5)	3.7 ± 0.6	3.5 ± 0.0	3.5 ± 0.5	3.3 ± 0.5	0.5938	0.9899	0.5938
HAW (cm)	149.0 ± 2.2	131.2 ± 7.6	149.2 ± 4.9	128.7 ± 6.4	<0.0001	0.6374	0.5854
HH (cm)	156.7 ± 3.8	134.3 ± 6.5	159.3 ± 5.2	136.2 ± 6.4	<0.0001	0.3582	0.8620
BL (cm)	177.7 ± 10.1	165.5 ± 8.5	172.0 ± 6.5	163.8 ± 5.3	0.0069	0.2900	0.5599
HG (cm)	208.2 ± 4.9	194.3 ± 10.1	209.0 ± 6.7	199.7 ± 12.3	0.0075	0.4375	0.5696
CBC (cm)	23.5 ± 1.3	19.8 ± 1.1	23.5 ± 1.0	20.7 ± 1.3	<0.0001	0.2550	0.2550
CD (cm)	78.8 ± 2.7	73.3 ± 3.5	77.0 ± 5.2	73.0 ± 3.3	0.0078	0.5081	0.6459
SC (cm)	38.8 ± 1.4	34.5 ± 1.9	38.8 ± 1.4	35.8 ± 2.2	<0.0001	0.3322	0.3322
TV (cm³)	945.2 ± 223.4	757.3 ± 123.5	1,114.7 ± 169.4	769.1 ± 198.9	0.0031	0.2670	0.3324
STT (mm)	0.7 ± 0.1	0.6 ± 0.1	0.8 ± 0.1	0.6 ± 0.1	0.0035	0.6548	0.9489
Skin color (%)	97.0 ± 5.0	97.0 ± 5.0	96.1 ± 5.6	93.9 ± 10.2	0.6252	0.4654	0.6252
ST (mm)	1.3 ± 0.1	1.1 ± 0.1	1.3 ± 0.1	1.1 ± 0.1	0.0002	0.5935	0.6785
HG/BW	0.2 ± 0.0	0.3 ± 0.0	0.3 ± 0.0	0.4 ± 0.0	<0.0001	0.1264	0.4870
HD (x10³)	703.0 ± 96.9^b^	713.5 ± 120.6^ab^	810.5 ± 166.3^a^	767.7 ± 195.8^ab^	0.8066	0.2292	0.0069
HL (mm)	7.5 ± 2.0^a^	8.0 ± 0.8^a^	4.5 ± 0.8^c^	6.3 ± 1.0^b^	<0.0001	<0.0001	<0.0001
CIE L*	55.8 ± 9.6	41.5 ± 7.1	67.9 ± 3.1	45.0 ± 5.5	<0.0001	0.0146	0.1575
CIE A*	1.4 ± 0.3	5.9 ± 2.1	1.8 ± 0.3	6.0 ± 1.9	<0.0001	0.6946	0.8360
CIE B*	6.9 ± 2.2^b^	10.5 ± 4.4^a^	11.3 ± 0.8^a^	10.5 ± 4.3^a^	0.3273	0.1336	<0.0001
CIE C*	7.0 ± 2.2^b^	12.1 ± 4.8^a^	11.4 ± 0.8^a^	12.2 ± 4.6^a^	0.0722	0.1559	<0.0001
CIE h	78.2 ± 2.0^b^	59.5 ± 4.9^c^	81.0 ± 1.8^a^	58.7 ± 4.5^c^	<0.0001	0.4986	<0.0001

BW: body weight; BCS: body condition score; HAW: height at withers; HH: hip height; BL: body length; HG: heart girth; CBC: Cannon bone circumference; CD: chest depth; SC: scrotal circumference; TV: testicular volume; STT: scrotal skinfold thickness; ST: body skinfold thickness; HG/BW: Heart girth/body weight ratio; HD: hair coat density; HL: hair coat length; CIE L*: lightness; CIE A*: Chromatic channel a* (red-green color intensity); CIE B*: Chromatic channel b* (yellow-blue color intensity); CIE C*: chroma; CIE h: hue angle

Notes: CIE L*, a*, b*, and derived variables C* and h correspond to the standardized coordinates of the CIELAB color space. The asterisk is part of the official notation and was originally adopted to distinguish the CIELAB system from the older Hunter Lab system; it does not indicate statistical significance

Different lowercase letters indicate significant differences (*P* < 0.05) among breed × season combinations only when the breed × season interaction was significant. SD: standard deviation

### Fresh and post-thaw semen

For fresh semen traits, there were no significant seasonal or breed differences (Table 4). On the other hand, sperm kinematics in post-thaw semen changed significantly between breeds and seasons (Table 5).

**Table 4:** Fresh semen traits (mean ± SD) of Pantaneiro and Nelore bulls in dry and rainy season

Parameters	Dry season		Rainy season		P-Breed	*P*-Season	*P*-Breed*Season
	Nelore (*n* = 6)	Pantaneiro (*n* = 6)	Nelore (*n* = 6)	Pantaneiro (*n* = 6)			
Motile (%)	81.7 ± 8.4	84.2 ± 7.5	81.4 ± 8.7	82.2 ± 6.5	0.4381	0.6036	0.6966
Vigor (0–5)	3.3 ± 0.6	3.5 ± 0.6	3.4 ± 0.8	3.6 ± 0.5	0.2487	0.6963	0.8146
Major defects (%)	7.4 ± 6.6	9.8 ± 9.3	5.4 ± 5.2	7.6 ± 5.9	0.3004	0.3264	0.9866
Minor defects (%)	3.2 ± 5.9	0.9 ± 1.3	1.6 ± 1.8	2.8 ± 4	0.7351	0.9452	0.2265
Total defects (%)	10.6 ± 6.8	10.7 ± 5.2	7.1 ± 6.2	10.4 ± 8.2	0.8073	0.7275	0.2604
LSIA (%)	86.9 ± 14.2	92.3 ± 6.7	83.8 ± 14.8	88.6 ± 7.7	0.3143	0.4400	0.8479
LSRA (%)	2.3 ± 3.3	3.0 ± 3.0	1.8 ± 2.5	2.1 ± 2.6	0.4781	0.4733	0.8808
DSIA (%)	4.0 ± 3.3	3.0 ± 4.9	5.5 ± 4.7	7.3 ± 6.7	0.7716	0.0555	0.3565
DSRA (%)	6.8 ± 13.4	1.7 ± 2.0	8.8 ± 14.1	1.9 ± 1.5	0.1612	0.8434	0.8887

LSIA: live sperm with intact acrosome; LSRA: live sperm with reacted acrosome; DSIA: dead sperm with intact acrosome; DSRA: dead sperm with reacted acrosome. SD: standard deviation

**Table 5:** CASA-based sperm kinematics and morphological traits (mean ± SD) of post-thaw semen of Pantaneiro and Nelore bulls in dry and rainy season

Parameters	Dry season		Rainy season		P-Breed	*P*-Season	P-Breed*Season
	Nelore (*n* = 6)	Pantaneiro (*n* = 6)	Nelore (*n* = 6)	Pantaneiro (*n* = 6)			
Motile (%)	30.9 ± 18.2	43.2 ± 14.3	34.2 ± 13.0	44.9 ± 19.7	0.0443	0.6515	0.8900
Progressive (%)	16.9 ± 10.1	26.1 ± 9.0	16.2 ± 7.1	22.6 ± 11.1	0.0205	0.5131	0.6583
Rapid (%)	19.9 ± 12.1	31.9 ± 12.0	21.2 ± 8.1	30.3 ± 12.7	0.0129	0.9636	0.7193
Slow (%)	14.7 ± 8.1^b^	18.7 ± 6.6^a^	17.7 ± 3.6^ab^	16.5 ± 2.5^ab^	0.2314	0.7283	0.0272
Static (%)	54.4 ± 24.5	38.2 ± 19.2	48.1 ± 14.4	37.7 ± 22.2	0.0495	0.6016	0.6488
VAP (µm/s)	65.6 ± 4.7	70.8 ± 10.2	58.4 ± 5.8	64.4 ± 6.2	0.0147	0.0039	0.8382
VSL (µm/s)	54.9 ± 4.4	58.8 ± 7.3	45.6 ± 5.6	49.1 ± 4.4	0.0229	<0.0001	0.8699
VCL (µm/s)	108.7 ± 9.1	119.0 ± 20.8	105.4 ± 15.4	116.6 ± 16.6	0.0361	0.5628	0.9289
ALH (µm)	0.1 ± 0.0	0.1 ± 0.0	0.1 ± 0.0	0.1 ± 0.0	0.8269	0.3918	0.9429
BCF (Hz)	29.0 ± 4.3	30.1 ± 4.0	26.7 ± 3.8	29.0 ± 1.9	0.1212	0.1162	0.5710
STR (%)	83.8 ± 2.9	83.1 ± 4.4	76.9 ± 9.0	75.8 ± 5.6	0.5871	0.0002	0.8947
LIN (%)	52.8 ± 4.8	51.8 ± 4.8	45.1 ± 7.7	44.2 ± 5.7	0.5947	0.0002	0.9734
Major defects (%)	14.2 ± 10.5	17.9 ± 10.4	12.1 ± 8.2	17.5 ± 6.8	0.2145	0.7046	0.7858
Minor defects (%)	5.6 ± 8.9	1.3 ± 1.4	2.4 ± 2.4	1.1 ± 1.0	0.1752	0.4068	0.4917
Total defects (%)	19.8 ± 14.9	19.2 ± 10.8	14.4 ± 10.1	18.6 ± 7.1	0.6702	0.5191	0.6087

VAP: average path velocity; VSL: straight-line velocity; VCL: curvilinear velocity; ALH: amplitude of lateral head displacement; BCF: beat cross frequency; STR: Straightness; LIN: Linearity.

Different lowercase letters indicate significant differences (*P* < 0.05) among breed × season combinations only when the breed × season interaction was significant. SD: standard deviation

The percentages of motile, progressive, VAP, and rapid sperm were higher for Pantaneiro bulls in both seasons (*P* < 0.05; Table 4). VSL was higher for Pantaneiro bulls in the dry season (*P* < 0.05), but there was no significant difference in the rainy season. There was no effect of season on VCL, but it was lower for Nelore bulls in the rainy season (*P* < 0.05). STR and LIN did not differ between breeds, however it reduced by about 10% in the rainy season compared to the dry season for both breeds. There was no effect of season or breed on total and major sperm abnormalities, while there was a greater percentage of minor defects for Nelore bulls during the dry season (*P* < 0.05).

The percentages of sperm with intact plasma membrane and acrosome (IMIA) and intact plasma membrane (IM) were comparable across the two staining procedures, as anticipated, demonstrating the reliability of the assays (Table 6). Pantaneiro bulls presented significantly higher percentages of intact cells than Nelore bulls in both seasons. Season and breed influenced the frequency of cells with intact membranes and with high mitochondrial membrane potential (JC-1a). The mean values of three cell categories, intact acrosome, intact plasma membrane, and low mitochondrial membrane potential (JC-1 d), reacted acrosome, intact plasma membrane, and low mitochondrial membrane potential (JC-1 g), and JC-1 h, were nearly zero.

**Table 6:** Viability of post-thaw sperm membranes and chromatin integrity (mean ± SD) of Pantaneiro and Nelore bulls in dry and rainy season

Parameters	Dry season		Rainy season		P-Breed	*P*-Season	P-Breed*Season
	Nelore (*n* = 6)	Pantaneiro (*n* = 6)	Nelore (*n* = 6)	Pantaneiro (*n* = 6)			
IMIA (%)	29.1 ± 14.8	41.3 ± 11.8	35.9 ± 11.5	47.6 ± 10.5	0.0039	0.0903	0.9406
DMDA (%)	29.3 ± 14.5	28.3 ± 15.8	21.5 ± 11	20.7 ± 6.1	0.8322	0.0789	0.9789
DMIA (%)	41.6 ± 20.9	30.2 ± 12.2	42.3 ± 10.4	31.3 ± 10.1	0.0170	0.8333	0.9746
IMDA (%)	0.1 ± 0.5	0.2 ± 0.5	0.3 ± 1.0	0.3 ± 0.8	0.8744	0.4327	0.8744
IM (%)	29.3 ± 15.0	40.7 ± 11.4	35.1 ± 10.3	46.7 ± 10.4	0.0051	0.1226	0.9791
DM (%)	70.7 ± 15.0	59.3 ± 11.4	64.9 ± 10.3	53.3 ± 10.4	0.0051	0.1226	0.9791
JC-1 a (%)	34.2 ± 18.1	41.4 ± 11.2	33.6 ± 11.9	51.8 ± 11.2	0.0084	0.2746	0.2253
JC-1 b (%)	11.9 ± 9.7	12.9 ± 12	14.7 ± 10.2	10.1 ± 7.2	0.5170	0.9920	0.3176
JC-1 c (%)	22.0 ± 20.7	20.1 ± 18.6	19.9 ± 12.7	10.7 ± 6.8	0.2320	0.2232	0.4300
JC-1 e (%)	24.7 ± 16.2	18.9 ± 9.9	23.2 ± 10.4	20.1 ± 5.9	0.2603	0.9603	0.7388
JC1- f (%)	6.8 ± 6.1	6.6 ± 5.4	8.2 ± 3.4	7.4 ± 3.3	0.7157	0.4572	0.8629
SIC (%)	91.1 ± 16.6	98.2 ± 3.2	98.8 ± 1.4	98.5 ± 1.6	0.4359	0.3559	0.3913
SDC (%)	8.9 ± 16.6	1.8 ± 3.2	1.2 ± 1.4	2.0 ± 2.6	0.5057	0.4180	0.3356

IMIA: Intact plasma membrane and acrosome; DMDA: Damaged plasma membrane and acrosome; DMIA: Damaged plasma membrane and intact acrosome; IMDA: Intact plasma membrane and damaged acrosome; IM: Intact plasma membrane; DM: Damaged plasma membrane; JC-1 a: Intact acrosome, intact plasma membrane and high mitochondrial membrane potential; JC-1 b: Damaged acrosome, damaged plasma membrane and low mitochondrial membrane potential; JC-1 c: Intact acrosome, damaged plasma membrane and low mitochondrial membrane potential; JC-1 e: Intact acrosome, damaged plasma membrane and high mitochondrial membrane potential; JC-1 f: Damaged acrosome, damaged plasma membrane and high mitochondrial membrane potential; SIC: Sperm with intact chromatin; SDC: Sperm with denatured chromatin

Different lowercase letters indicate significant differences (*P* < 0.05) among breed × season combinations only when the breed × season interaction was significant. SD: standard deviation

For Pantaneiro bulls, sperm with intact chromatin (SIC) did not differ across seasons; however, it decreased significantly in Nelore bulls during the dry season. Sperm with denatured chromatin (SDC) was significantly higher in the dry season for Nelore bulls, but did not differ for Pantaneiro bulls within or between seasons.

### Correlations

In the dry season, air temperature and THI had significant negative correlations with total sperm defects in fresh semen. At the same time, they were positively correlated with intact plasma membrane and damaged acrosome (IMDA). In post-thaw semen, THI showed a positive correlation with STR. In contrast, MRT was negatively correlated with sperm motility and JC1-a, and positively correlated with static cells, intact acrosomes, damaged plasma membranes, and low mitochondrial membrane potential (JC1-c). External morphological traits were positively correlated with static cells, damaged plasma membrane and intact acrosome (DMIA), and intact acrosome, damaged plasma membrane and high mitochondrial membrane potential (JC1-e), while was negatively correlated (*P* < 0.05) with sperm defects, IMIA, IM, JC1-a, and post-thaw sperm kinetics, with the exception of STR, which was positively correlated with BL and HG. Skin and hair coat colors were positively correlated with sperm motility in fresh semen, while they were negatively correlated with sperm defects. Hair density (HD) showed a negative correlation with sperm defects, JC1-b, and JC1-d, while it was positively correlated with JC1-e, IMDA, DMIA, and normal cells. Regarding CASA parameters, sperm velocities were positively correlated with hair coat color (a*, b*, and c*), ALH was positively correlated with lightness (L*), b*, and c*, while BCF was negatively correlated with L*, b*, c*, and h*. Motile, progressive, and rapid sperm showed a negative correlation with L* and h*.

In the rainy season, sperm abnormalities in fresh semen were positively correlated with THI. Body size, SC, TV, and STT were negatively correlated with sperm defects, sperm velocities, IMIA, DMDA, IMDA, IM, and JC1-a. In contrast, they were positively correlated with STR, LIN, slow cells, DMIA, DM, JC1-b, and JC1-c. Skin color showed a negative correlation with sperm motility in fresh semen, IM, DMDA, IMDA, and JC1-b, while showing a positive correlation with VCL. Coat color traits were negatively correlated with sperm motility, progressive motility, rapid sperm, IMIA, IM, live sperm with intact acrosome (LSIA), and JC1-a, while they were positively correlated with dead sperm with reacted acrosome (DSRA), static sperm, DM, JC1-b, and intact DNA.

**Fig. 1:**
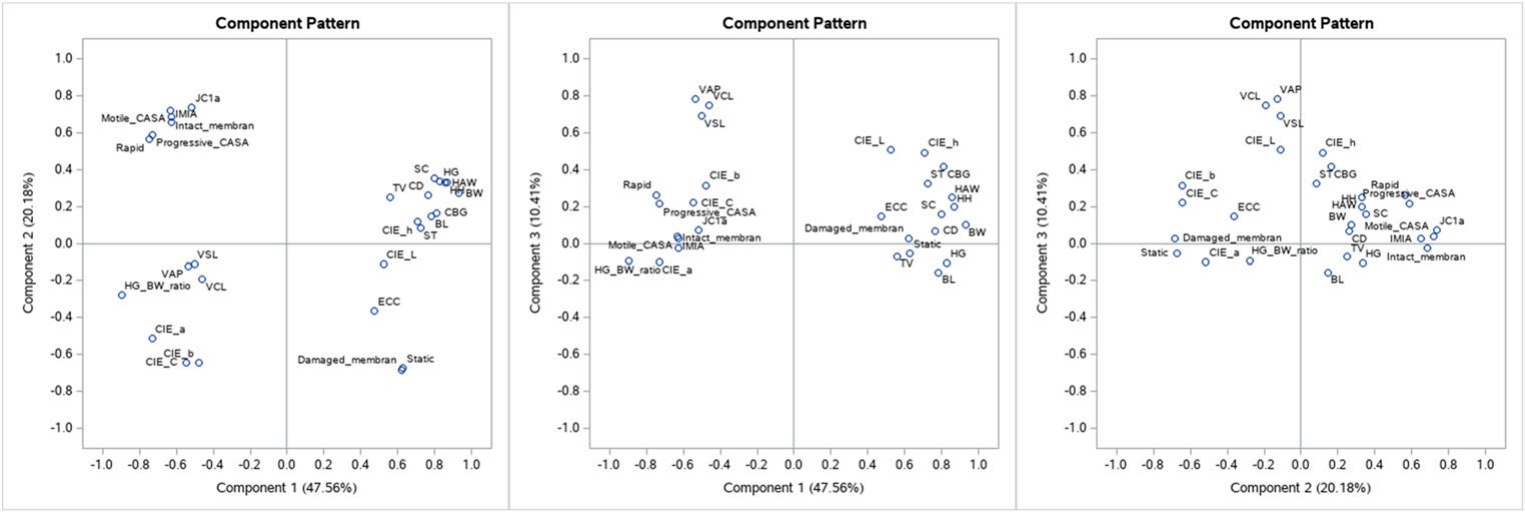
Component pattern plots for the first three principal components in the dry season. (**A**) Component 1 vs. Component 2. (**B**) Component 1 vs. Component 3. (**C**) Component 2 vs. Component 3

**Fig. 2:**
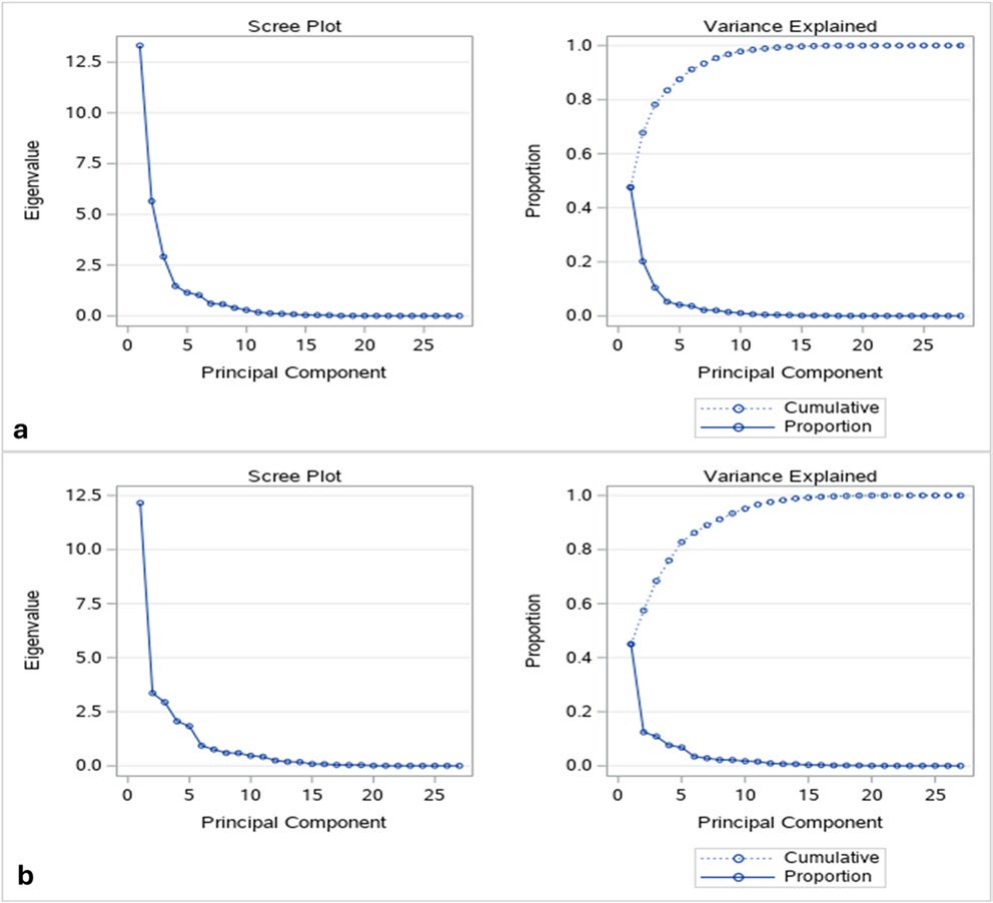
Scree plot and variance explained plot for the PCA conducted in the dry (**A**) and rainy (**B**) seasons. These plots indicate the eigenvalues of each component and the proportion of total variance retained across components

With respect to the results of PCA in the dry season, the eigenvalues of the correlation matrix of PCA showed that the first three principal components together account for 78% of the variance in the observed variables (Fig. 1); this is depicted on the variance explained plot (Fig. 2), indicating that they provide a good summary of the data. Each subsequent component accounts for less than 5%. The scree plot (Fig. 2) shows that the eigenvalue of the first component is approximately 13, and that of the third component has decreased to nearly 2.5. This confirms the existence of the three common components.

**Fig. 3:**
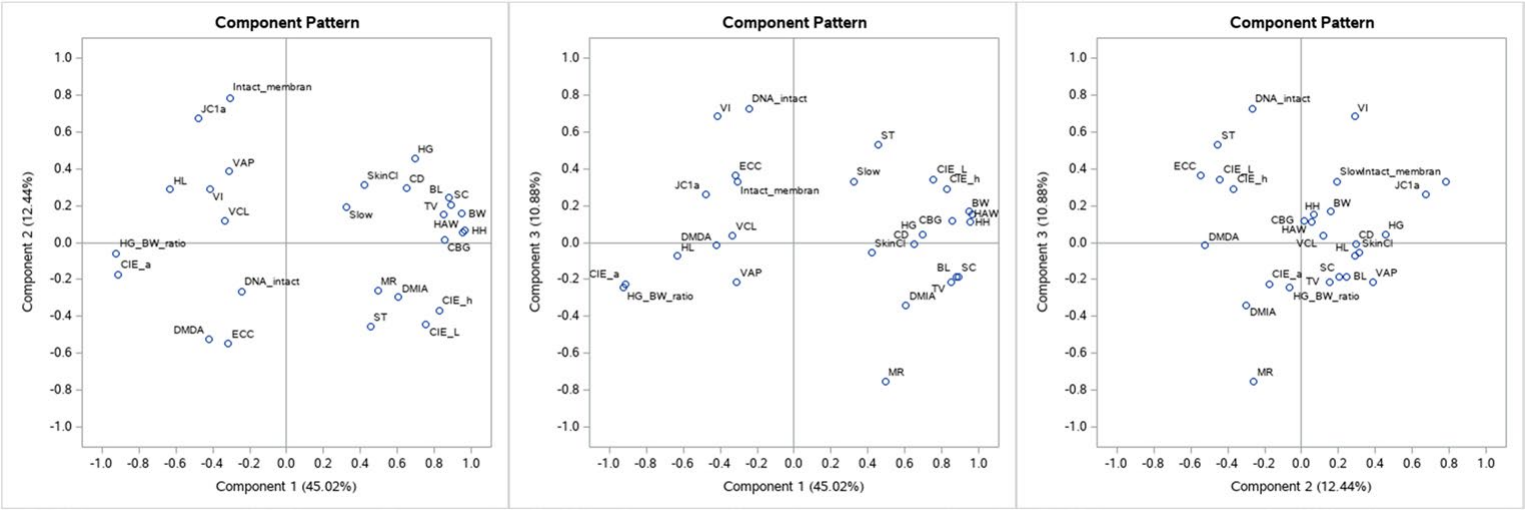
Component pattern plot for the first three principal components in the rainy season, illustrating how the main contributing variables differ from the dry-season structure. (**A**) Component. 1 vs. Component 2. (**B**) Component 1 vs. Component 3. (**C**) Component 2 vs. Component 3

For the rainy season, the first four principal components account for 76% of the total variance (Fig. 3), which is also illustrated in the variance explained plot (Fig. 2). The scree plot (Fig. 2) shows that the eigenvalue of the first component is approximately 12 and the eigenvalue of the fourth component is decreased to under 2.0. This helps to confirm the existence of the four common components.

## Discussion

Our findings suggest that smaller bulls may be better suited to hotter environments, and Pantaneiro bulls showed an advantage over Nelore bulls in this context. Although these relationships are based on correlations and therefore cannot establish causality, they align with well-described biophysical principles. Smaller animals have a higher surface-area-to-volume ratio, which enhances heat dissipation, and energy expenditure in mammals scales positively with body mass (Cabezas-Garcia et al. 2021). Together, these physiological considerations help explain the observed patterns.

The THI data indicated that bulls experienced heat stress in both seasons (Thom 1959; Hahn 1985); however, the index remained within the alert and danger zones for longer during the rainy season than during the dry season. Lower THI found in the dry season resulted from the inverse relationship observed between AT and RH, which in turn facilitates the heat exchange with the environment and, consequently, contributes to the maintenance of the animal’s thermal comfort. Despite their wide thermal tolerance, cattle still experience heat stress, and less-adapted animals may experience significant thermal discomfort or even die if the temperature regulation system cannot compensate. In extensive cattle production, metabolism accounts for nearly one-third of total heat load, and animals’ capacity to eliminate metabolic heat efficiently is crucial for maintaining stable body temperature (Shephard and Maloney 2023). Many factors can affect an animal’s response to the thermal environment, such as body size, hair and coat characteristics, sex, genotype, temperament, health, adaptation, and age. The complex interaction between an animal’s physiology and behavior, and the mechanisms of heat transfer, leads to heat stress. In addition, animal behavior is an extra dynamic element included in these calculations for heat balance (Dos Santos et al. 2021).

Correlations found in this study reinforce the theory that the animal’s external morphology, including size, is also associated with spermatic characteristics. Spermatic kinetics and intact membranes showed negative correlations (*P* < 0.05) with variables related to animal size, testes skinfold thickness, body skin thickness, skin surface area-to-volume ratio, hair length, and skin color. In other words, smaller animals, with thin skin and short, reflective hair, tend to present higher values for spermatic kinetics and viability. Pantaneiro bulls have all these characteristics, except for the lower reflectance of the hair coat in comparison with Nelore. However, other adaptive traits in this breed appear to compensate for this disparity (McManus et al. 2005). Cesca et al. (2021) found that beef cattle in the Brazilian state of Mato Grosso do Sul (MS) are subjected to highly stressful environmental circumstances. Therefore, it is important to study breeds adapted to extreme climatic conditions, such as the Pantaneiro, to maintain cattle breeding systems and, consequently, the production of meat and milk.

Both Nelore and Pantaneiro bulls showed good quality of fresh semen, with no season or breed effects on sperm kinetics, sperm defects, or membrane viability. On the other hand, breed and season influenced the kinetic characteristics of post-thaw semen. Pantaneiro bulls showed better parameters than Nelore, particularly during the hotter season, but semen quality remained within satisfactory parameters across seasons (CBRA 2013). This result reinforces the idea of an animal of European origin well adapted to adverse climatic conditions (Vieira et al. 2022) and thus preventing major drops in seminal quality.

Malama et al. (2017) and Llamas-Nucenõ et al. (2020) studied Dutch bulls and observed lower values for all spermatic-kinetics characteristics during the hot and humid season, indicating reduced adaptation of European breeds to certain climates. The authors considered that sperm with STR and VAP above 70% and 50 μm/s, respectively, could be classified as agile, as in the present work. This observation is consistent with the study’s results and supports the assumption that the locally adapted Pantaneiro breed outperformed Nelore bulls.

The results of Malama et al. (2012) may indicate an effect of the month and climatic factors (*P* < 0.05) on the variation of spermatic DNA fragmentation among bulls (*Bos taurus taurus*). The authors suggest that climatic fluctuations may disrupt sperm chromatin stability during the maturation phase. Previous research has also found that the individual effect of the bull, rather than breed or season, is more important in driving changes in sperm chromatin fragmentation (Karoui et al. 2012; Souto et al. 2022). Thus, to identify bulls with abnormal DNA during the breeding soundness evaluation, it is helpful to perform a sperm chromatin integrity assay. Nava-Trujillo et al. (2011) found an average of 4.2% sperm cells with denatured chromatin in cryopreserved semen of Brahman bulls using the toluidine blue staining. Holstein bulls reared in a mild climate, with an average summer temperature of 20.5 °C, showed a higher rate of chromatin fragmentation (Sabés-Alsina et al. 2017). In a three-year study of Nelore bulls reared in the Brazilian Central-West region, approximately 9% denatured chromatin was observed in fresh semen (Addad et al. 2009). However, there was no impairment in semen quality or fertility rates across the reproductive seasons. In another study involving more than 1,000 semen doses from 201 Holstein bulls, a 7%-10% rate of sperm DNA fragmentation was associated with low fertility (Karoui et al. 2012). In this study, regardless of the season, the percentage of DNA fragmentation in the Pantaneiro was lower than in the reported works (Addad et al. 2009; Nava-Trujillo et al. 2011; Karoui et al. 2012) and even lower than in the Nelore during the dry season.

The percentage of intact cells (MIAI and MI) and intact cells with high mitochondrial membrane potential (JC1a) differed significantly between breeds and seasons. In general, these categories increased during the rainy season, and the highest intact membrane values were observed in Pantaneiro bulls. Contrary to this study, Malama et al. (2017) reported more intact cells in winter, which corresponds to the dry season in this study. The authors observed that season affected motility, the composition of sperm plasma membrane lipids, and intracellular calcium concentration, which can lead to a premature acrosome reaction, concluding that seminal quality was lower in summer.

The season effect on seminal characteristics tends to be more pronounced in *Bos taurus* bulls due to adaptive differences in relation to the *Bos indicus* subspecies. As an example, the presence of genes for thermotolerance (Hansen 2004), greater number of proteins related to motility and fertility (Ashrafzadeh et al. 2013) and the differences in the vascular anatomy of *Bos indicus* testes (Brito et al. 2004), are part of the adaptation to hot climates which improve body and testicular thermoregulation. Additionally, sperm from *Bos taurus* have a higher concentration of polyunsaturated fatty acids in the plasma membrane than those from *Bos indicus*, making them more vulnerable to oxidative stress (Rodrigues et al. 2015). However, these characteristics cannot yet be extrapolated to locally adapted *Bos taurus* breeds.

Adverse environmental conditions, especially elevated temperatures, can affect the dynamics of lipids that make up the plasma membrane, altering its fluidity and morphology and compromising its integrity. In Holstein bulls, the lipid composition of spermatozoa tails varies seasonally (Argov-Argaman et al. 2013), with lower concentrations of polyunsaturated fatty acids and cholesterol during summer. In addition, the decrease in lipid concentration in tail membranes was associated with decline in motility and sperm velocity. However, other factors not evaluated in this study may also contribute to seasonal variation in sperm membrane quality. Nutritional conditions - particularly pasture availability and quality, as well as variations in nutrient intake, digestion, and absorption — are known to influence reproductive physiology in bulls and can vary markedly between seasons in extensive systems. Although these aspects were not measured here and therefore cannot be linked to our findings, we mention them as plausible contextual factors reported in the literature.

The findings of this study suggest that factors other than weather are more likely to explain the increased viability of sperm membranes during the rainy season. Although the THI was lower in the dry season, the reduced quantity and lower quality of pasture may have adversely affected membrane stability. THI increased during the rainy season, along with increased food availability, which may have improved sperm membrane quality.

The proportion of intact membranes across both seasons showed that Nelore bulls consistently had lower values than Pantaneiro bulls. Despite being acclimated to the tropics, Nelore also underwent a rigorous genetic selection process for high production (Oliveira et al. 2002; Rodrigues et al. 2022). Pantaneiro bulls presumably require less nutrition because of their smaller size and the fact that they have not undergone the same intense genetic selection as commercial breeds. This may partially explain why the season had a lower impact on the percentage of intact membranes in the semen of Pantaneiro bulls.

Based on these findings, we concluded that the quality of fresh and cryopreserved semen from both tropic-adapted breeds varies with the season, particularly in sperm viability and kinetics, and that Pantaneiro bulls outperform Nelore bulls under the same conditions. Both breeds maintained acceptable semen quality under heat stress, but the Pantaneiro bulls stood out for their stability and overall performance. This reinforces the value of the Pantaneiro as a genetic resource as a *Bos taurus* breed well-adapted to tropical environments, it carries traits directly relevant to conservation strategies and future breeding programs focused on resilience and reproductive efficiency.

## Data Availability

The datasets generated during and/or analysed during the current study are available from the corresponding author on reasonable request.
